# Prof. Aston’s Introductory

**Published:** 1888-10

**Authors:** 


PROF. GASTON'S INTRODUCTORY.—We held back the present issue of the RECORD to give place to the "Introductory Address" of Prof. Gaston, delivered at the opening of the SOUTHERN MEDICAL COLLEGE, in the College Auditorium, at 11 o'clock, on the 2d of October. It will be found in the original department of this number, and is an able address, interesting and eminently practical. His remarks on Listerism may be regarded as expressive of the views of older and more conservative surgeons both here and across the water. It is perhaps well that men are found amongst us who, while progressive and practical in the profession, serve to hold a check-rein upon those whose enthusiasm leads to the too ready praise of all that is new and to the disparagement and desertion of the old and long established agencies and methods in the practice.

The opening of the College was in all respects a decided success, the class being a large and intelligent one, there being present also a number of citizens and visitors who manifested marked interest in the exercises.

The opening of the Dental Department took place at the Lecture Hall, on Walton street, at 4 p. m. Prof. T. S. Powell, the President, addressed the class in kind and admonitory words. This was followed by a very able and instructive address by L. D. Carpenter, D. D. S., Prof. of Pathology in the Dental Department. A goodly number of students were present and the opening was in all respects encouraging.

